# Successful Outcome of a Case of Acute Myeloid Leukemia with t(8;21)/AML-ETO Following Langerhans Cell Histiocytosis

**DOI:** 10.4274/tjh.galenos.2019.2019.0126

**Published:** 2019-11-18

**Authors:** Guangqiang Meng, Jingshi Wang, Jiancheng Huang, Yini Wang, Na Wei, Zhao Wang

**Affiliations:** 1 Beijing Friendship Hospital, Capital Medical University, Beijing, China

**Keywords:** Langerhans cell histiocytosis, Acute myeloid leukemia, Allogeneic hematopoietic stem cell transplant

## To the Editor,

The occurrence of Langerhans cell histiocytosis (LCH) and acute myeloid leukemia (AML) in the same case has been reported occasionally. We report a new case of AML with t(8;21)/AML-ETO in an adolescent after LCH. To our knowledge, this is the first description of AML with t(8;21)/AML-ETO after LCH diagnosis and therapy.

A 15-year-old boy was diagnosed with LCH in October 2010. He presented with a 1-year history of a skull mass. After 9 cycles of ifosfamide, vincristine, etoposide, and prednisone, the skull mass disappeared. Two years later, the patient presented to the Hematology Department of Beijing Friendship Hospital with progression of his disease in the form of lumber fracture. The mutation BRAF V600E was negative. After relapse of LCH, he received 6 cycles of etoposide and prednisone and 1 cycle of etoposide, prednisone, cyclophosphamide, and vincristine. On 12 March 2013, he received an autologous hematopoietic stem cell transplant. When he came to the clinic with complaints of dizziness on 20 November 2017, a routine blood examination was performed with the following results: white blood cell count, 6.3x10^9^/L; hemoglobin, 60 g/L; and platelet count, 12x10^9^/L. Bone marrow biopsy showed 69% myeloblasts, and Auer rods were found. The immunophenotype profile of the blast cells was CD34 (++), CD13 (+), CD33 (++), CD117 (++), CD38 (+), CD15 (+), HLA-DR (++), MPO(+). Cytogenetic analysis revealed 46, XY, t(8;21)(q22;q22)[20]. The *AML-ETO* and *WT1* genes were positive. The patient responded well to induction chemotherapy. Standard DA chemotherapy (daunorubicin and cytarabine) was given and the boy achieved complete response (CR) after one cycle. After an additional cycle of DA consolidation chemotherapy, he received an HLA-identical sibling allogeneic hematopoietic stem cell transplant (HSCT). He received a conditioning protocol composed of busulphan and cyclophosphamide, and he was given fluconazole and acyclovir as infection prophylaxis and cyclosporine and mycophenolate mofetil as graft-versus-host disease prophylaxis. Up to 30 March 2019, the patient was in a state of persistent CR for 16 months after the diagnosis of the AML, and the *AML-ETO* and *WT1* genes were negative.

There is an association between LCH treatment and subsequent development of AML; however, the reason why AML develops in patients treated for LCH is not entirely understood. The chemotherapy for LCH and genetic predisposition may be explanations. Previously, 27 such cases have been reported [1], and lymphomas, solid tumors, and other hematologic malignancies have been associated with chemotherapy for LCH [2]. Most patients develop AML at least 2 years (mean: 5.5 years) after LCH treatment [3]. LCH treatment agents, together with the genetic predisposition of the patient, might therefore be the reason for AML development. Etoposide, a DNA-topoisomerase II inhibitor, is commonly employed in LCH treatment and is primarily related to therapy-related AML (t-AML) [4]. A safe dose of etoposide does not truly exist; between 2 and 20 years after exposure to etoposide, 1%-5% of patients may develop t-AML [5]. Etoposide-associated AML has been described after its usage for a wide spectrum of diseases, including non-Hodgkin lymphoma [6], acute lymphoblastic leukemia [7], solid tumors [2], and hemophagocytic lymphohistiocytosis [5]. The cytogenetic abnormalities of t-AML reported in patients with LCH include t(15;17), 11q23, +16, and +21 [8,9,10]. The finding of t(8;21)(q22;q22) in a t-AML patient with LCH has not been reported previously, although it was reported in t-AML patients with other malignant neoplasms, including solid tumors, lymphomas, and other hematologic malignancies [11]. Most of the t-AML cases with t(8;21) reported are t(8;21)(q22;q22); other breakpoints of t(8;21) are rare. However, the current findings indicate a worse outcome for t-AML with t(8;21) compared to de novo AML with t(8;21)(q22;q22) [11]. Throughout the treatment process, the case is more likely to be that of t-AML.

Additionally, previous studies have suggested common neoplastic precursors for LCH and AML [12]. Recent molecular analysis of human LCH samples and mouse models showed that the origin cell may be a myeloid-derived precursor [13]. Furthermore, genomic screening has revealed the presence of BRAF, ARAF, and somatic MAP2KI mutations in the majority of LCH and AML patients’ specimens [14,15]. Cases in which LCH occurred concurrently and after AML have also been reported [10,16,17]. Xu et al. [17] reported a case where LCH evolved into AML without chemotherapy including etoposide for LCH. Therefore, researchers have accepted the possibility of genetic predisposition to facilitate the development of pathogenic molecular abnormalities. Yohe et al. [10] reported four patients who presented with acute leukemia of myeloid or ambiguous lineage in association with LCH. One patient had trisomy 21 in both the leukemic blasts and LCH cells, indicative of a clonal relationship. Another patient expressed CD2, CD13, and CD117 on both the LCH cells and the leukemic blasts, suggesting a possible clonal relationship. These reports suggest that LCH and AML might have a common neoplastic stem cell.

In our case, successful allogeneic HSCT not only controlled the patient’s AML but also had a long-lasting effect on his relapsed LCH. For these patients, induction chemotherapy combined with allogeneic HSCT is a good choice.
